# Annotations

**Published:** 1894-07-14

**Authors:** 


### ANNOTATIONS.

### Is Cure Superfluous?

Two doctors of ultra-modern school were discussing the progress of their science the other day. One of them affirmed with enthusiasm the conviction that medical science, the knowledge of normal structure and function, the knowledge of aberrant processes and results, the whole, round, and complete knowledge of physical man, normal and abnormal, was becoming more perfect and more satisfying to the mind every day. The other agreed with equal enthusiasm, but ventured a mild plea for therapeutics, or rather, hinted that the "healing power" did not quite keep pace with the conquests of knowledge. "That," said the first, "is of small importance compared with the assured scientific gains." "Religion," he continued, "though no longer of much use as a practical instrument for the improvement of morals, is yet of perennial interest from a speculative and philosophical point of view." We leave the theologian to settle the question of the quantitative and qualitative value of religion in the region of practical morals. But we venture to protest with the utmost possible concentration of protesting energy against the doctrine that in medicine "mere cure" is of secondary importance, whilst the acquisition and perfecting of knowledge is the true aim and essence of medical science. Nay more, we urge that whenever, if ever, the inspiration of "cure" shall cease to be operative the enthusiasm for science will die and become extinct. It is man, after all, who is eternally interesting to man. The man, not of the physiologist only, but of the theologian and of the philosopher as well—man who thinks and feels, man who can be happy or miserable: and the highest function which medicine has to perform is, not the accumulation of knowledge, but the making of individual and universal man well and happy, and therefore successful and victorious throughout his life.

### The Bacillus Diphtheriæ in Cows' Milk.

For some little time there has been strife between Dr. Abbott, of Philadelphia, and Dr. Klein, of London, on the question of the possible spread of diphtheria by means of cows' milk. Dr. Klein, in a learned paper, considered that he had demonstrated the fact that the diphtheria bacillus could be obtained from the milk of a cow infected with diphtheria; that the bacillus so obtained could be cultivated externally, as, for example, on agar or gelatine; and that cultures so produced were deadly to guinea pigs. He held, therefore, that he had at least demonstrated the possibility of the spread of epidemics of diphtheria by means of cows' milk. Dr. Abbott, at this point, entered the field, and making experiments, as he said, similar in all or most respects to those made by Dr. Klein, failed to produce results which could in any proper sense be said to be confirmatory of those of Dr. Klein. Here was a charming contradiction in the region of "experimental" and "exact" science. Dr. Abbott made the most of his negative results, to the discrediting of Dr. Klein's scientific competency, and to the upheaval of his peace of mind. In the current number of the *Journal of Pathology and Bacteriology* Dr. Klein has a fresh innings, in which, in our judgment, he completely bowls Dr. Abbott out. He shows first of all that Dr. Abbott had not made his experiments on an identical, or even a similar, basis to his own; and, secondly, he advances the absolutely conclusive argument that negative results can never for a moment have the same weight as proved positive results. The conclusion of the matter—and this is really the one grain of wheat in several bushels of rather acrimonious scientific "chaff"—the definite conclusion is this, that cows inoculated with the human diphtheria bacillus develop local and general disease of a severe character, and that the milk of such cows does, in some instances at any rate, contain the bacillus diphtheriæ; and that the bacillus in the milk, when injected at once into guinea pigs, or cultivated and then injected, kills the guinea pigs. It seems then to be established beyond dispute that diphtheria may be spread by means of cows' milk. It is to be hoped, however, that we shall yet hear a good deal more of the matter, not only from Drs. Klein and Abbott, but also from other experimenters.

### Derby Sets an Example.

The opening of the County Infirmary at Derby by the Duke of Devonshire last week brought a great county enterprise to an honourable termination. Early in the Nineties the authorities of the hospital had to face the fact that their fine old building was hopelessly insanitary, and that the demands of modern science made a new building imperative. Two questions had to be faced: the question of money and the question of structure. The managers faced both; and faced them in such a way that they have set an example which may well be followed by all hospital managers in similar circumstances, whether in country or town. Their first resolve was that money enough should be spent to raise a building worthy alike of the science of the times and of the City and County of Derby. The building, when completed, will cost hardly less than £100,000, and of this vast sum the greater part has already been promised or paid. The question of structure presented almost greater difficulties. One of the easiest things in the world is to spend as much money as would build three good hospitals in rearing one bad one. The Derby managers have not split upon this rock. They have reared one of the completest and best equipped hospitals in the whole kingdom. The interesting question for other hospital managers to ask is, How has it all been done? The answer is one which Dr. Samuel Smiles would have been prompt to give. It has been done by means of that self-reliance and self-help which are so characteristic of the old-fashioned Englishman. Not only have the Derby people raised their own funds, but they have selected their own plans—an almost more critical venture—and they have superintended the carrying of them out to the last stone. The result is a hospital, which, in point of appearance, sanitary efficiency, and suitability for the manifold purposes of hospital life and work can hardly be surpassed. All other hospital managers who contemplate either rebuilding or repairing and renewing, should "go and do likewise."

